# Supplementary material for: Integrated, Automated, Fast PCR System for Point-Of-Care Molecular Diagnosis of Bacterial Infection
Source: Sensors (Basel). 2021 Jan 7;21(2):377. doi: 10.3390/s21020377 (PMC7827619; doi:10.3390/s21020377)
Supplement: Supplementary file 1 [file sensors-21-00377-s001.pdf]

Supplementary Information

Integrated, automated, fast PCR system for point-of-care molecular diagnosis of bacterial infection

Dongkyu Lee[1], *Deawook Kim*[1, 3], *Jounghyuk Han*[1, 3], *Jongsu Yun*[1], *Kang-Ho Lee*[1], *Gyu Man Kim*[3] *Ohwon Kwon*[1*], *and Jaejong Lee*[2*]

[1]Department of Medical Devices, Korea Institute of Machinery and Materials (KIMM), Daegu, 42994, Republic of Korea

[2]Nano-Mechanical Systems, Korea Institute of Machinery and Materials (KIMM), Daejeon, 34103, Republic of Korea

[3]Department of Mechanical Engineering, Kyungpook National University, Daegu, 41566, Republic of Korea

[*]Corresponding Author: owkwon@kimm.re.kr (O.W), jjlee@kimm.re.kr (J. L.)

Table S1. Protocols of sample preparation for both menual and automated methods

	Manual method	Automated method
**Cell preparation**	centrifugation of Salmonella cell(1*10^8 CFU) (1mL) at 9,000RPM	
	Removing top liquid on the cell pellet	
	Resuspension using 1x PBS solution(Corning Cat No. 21-040-CV)	
	Add Proteinase K (20µl) (Thermo fisher, Cat No. BP1700-100)	
**Lysis**	Add Lysis buffer (200µL)(Qiagen, Cat No. 67563), mixing and reacting for 5min	Add lysis buffer (200µl) and magnet bead(20 µl) and Isopropanol(300 µl) in a tube, and then, take 200 µl and transfer it to the lysis well and moving tool for shanking mix
**Bead binding**	Add magnet bead(Qiagen, Cat No. 1026883) 20 µl and 300 µl of isopropanol (Sigma-Aldrich, Cat No. I9516) and pipetting mix for 3min reaction.	
	Separating magnetic bead	Moving magnet tool to separating magnetic bead
	Remove top liquid in the tube	
		Moving magnet tool to Wash I buffer well
**WashI**	Add 800µl of Wash I buffer (Cosmogenetech, Cat No. CMB-007) and vortex mixing	Shanking mix (Wash I buffer 200 µl)
	Separating magnetic bead	Moving magnet tool to separating magnetic bead
	Remove top liquid in the tube	
		Moving magnet tool to Wash II buffer well
**WashII**	Add 800µl of Wash II buffer (Cosmogenetech, Cat No. CMB-005) and vortex mixing	Shanking mix (Wash II buffer 200µl)
	Separating magnetic bead	Moving magnet tool to separating magnetic bead
	Remove top liquid in the tube	
		Moving magnet tool to Elution buffer well
**Elution**	Add 500µl of Elution buffer(Qiagen, Cat No. 19077) 500ul	Shanking mix (Elution buffer 200µl)
	Separating magnetic bead	Remove magnetic bead
	Complete DNA extraction	Complete DNA extraction

- The commercial electronics and motors for the automated fast PCR system

Step motor (FL42STH33-0956A, Devicemart, Korea), Servo motor (HS-311, Hitec Rcd korea Inc., Korea), Step motor driver (A3967, twin chip), and liner bearings and slides (IGUS, Germany) were purchased to fabricate X-Z axis translational stages. Ceramic heater(CB1, Scipia, Korea) and Peltier cooler (TEC1-12706, SMG, Korea) were purchased to generate heating-cooling cycles of amplification chamber. Temperature variations were monitored by thermistor (Th310J39GBSN, Ampenol advanced sensor, USA). Super bright blue 3mm led (ada-301, Devicemart, korea), 466nm fluorescence filter (#86-341, Edmund optics, USA), 520nm fluorescence filter (#67-016, Edmund optics, USA), Raspberry Pi3 and camera were purchased for monitoring fluorescence images. The parts of the system were designed and printed by 3D printer (Stratasys uPrinter professional desktop 3D printer).

S1. Effects of sample volume on the thermocycle speed

The thermal cycle for samples of volume 20 μL completed 40 cycles in 12.8 min. The small reduction of amplification time is due to the heat transfer limitation by the conical microtube.

Figure S1. The thermal ramp cycles at the various sample volumes; 20 μL (black), 30 μL (red), 40 μL (blue), and 50 μL (green).

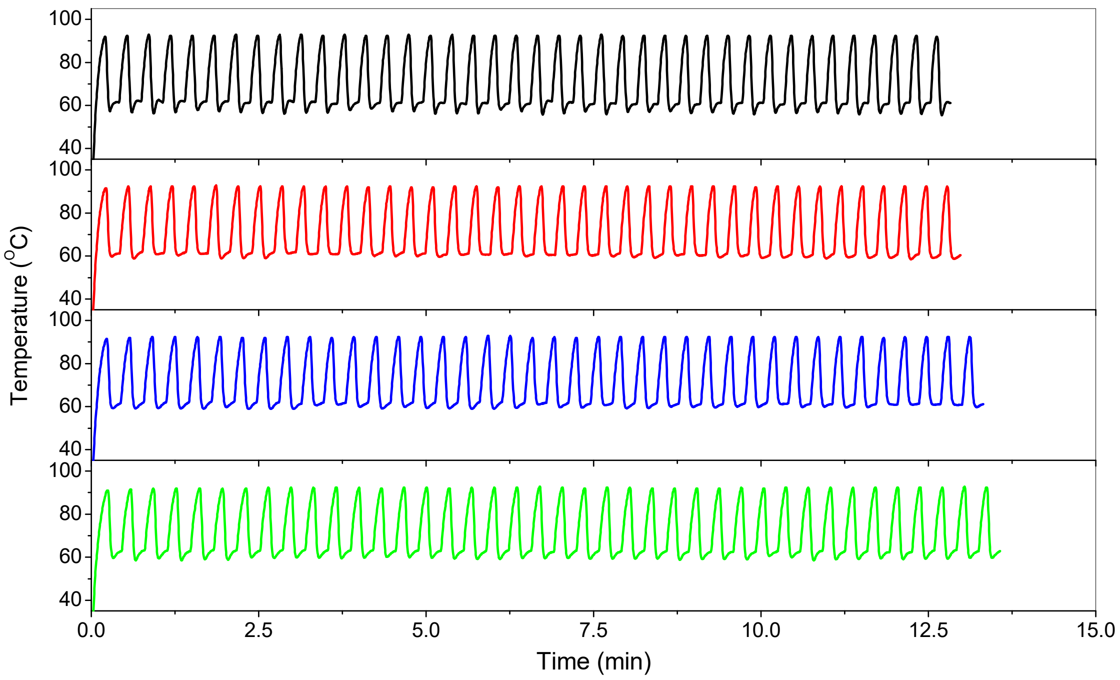

S2. Effects of heating block temperature on the fluorescence intensity

If the heating block temperature is over 105 °C, variations in the fluorescence intensity was decreased due to denaturation of Taq DNA polymerase.

Figure S2. variations in the fluorescence intensity depending on temperature of the heating block

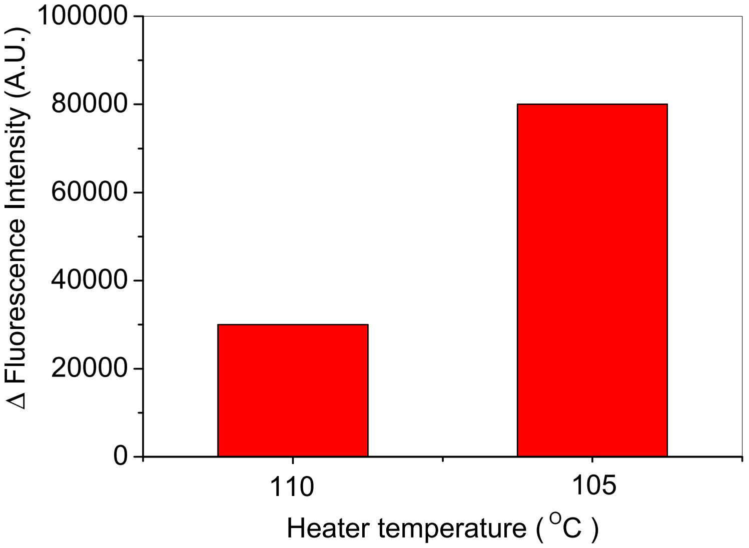

S3. Analysis of the fluorescence intensity from summing brightness of fluorescence images

The fluorescence image was monitored and analyzed by a single-board microcomputer (Raspberry Pi). The captured image was cut to a size of 280x300 pixels, and then total brightness value was calculated by summing all the brightness values of each pixel.

Figure S3. Methods of fluorescence image analysis; (a) Algorithm of image analysis in the control panel of a single-board microcomputer (Raspberry Pi), (b) a raw full fluorescence image, (c) greyscale of the full image,(d) a captured image of greyscale in a size of 280x300 pixels.

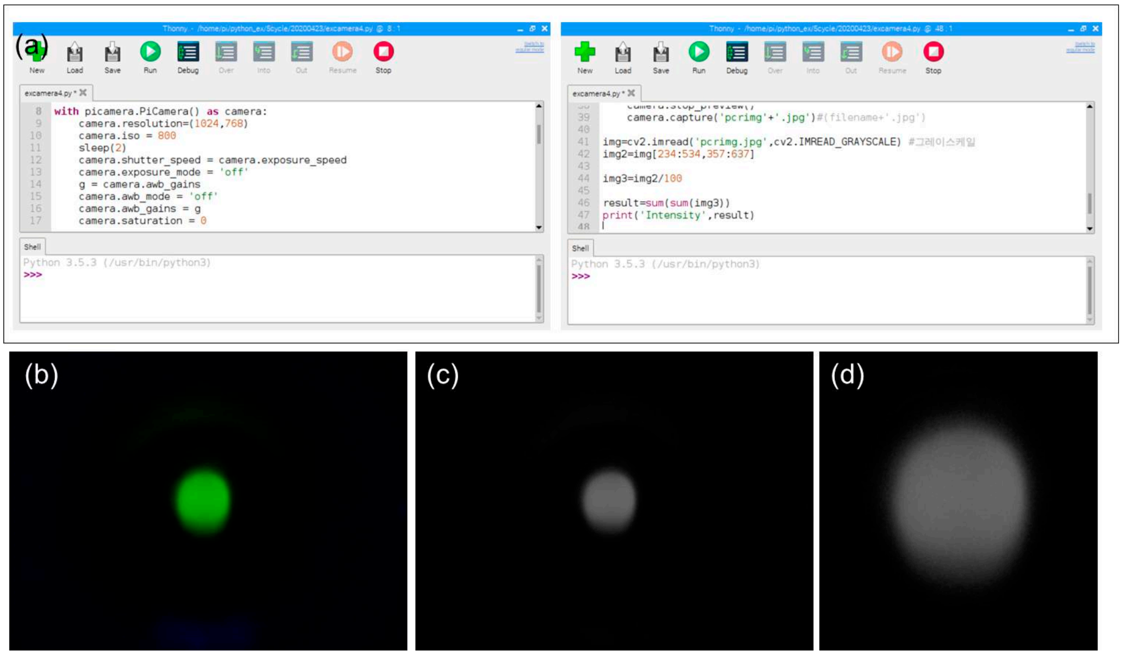

S4. Semi-logarithmic plot of Ct versus cycle number for the serial diluted samples.

As dilution increased, the Ct increased and the maximum change in fluorescence intensity at 40 cycles decreased in Figure 6a. The logarithm plot of DNA concentration versus Ct (Figure S4) is linear. The linear trend is generally utilized for quantification analysis.

Figure S4. Semi-logarithmic plot of Ct versus cycle number for the serial diluted samples

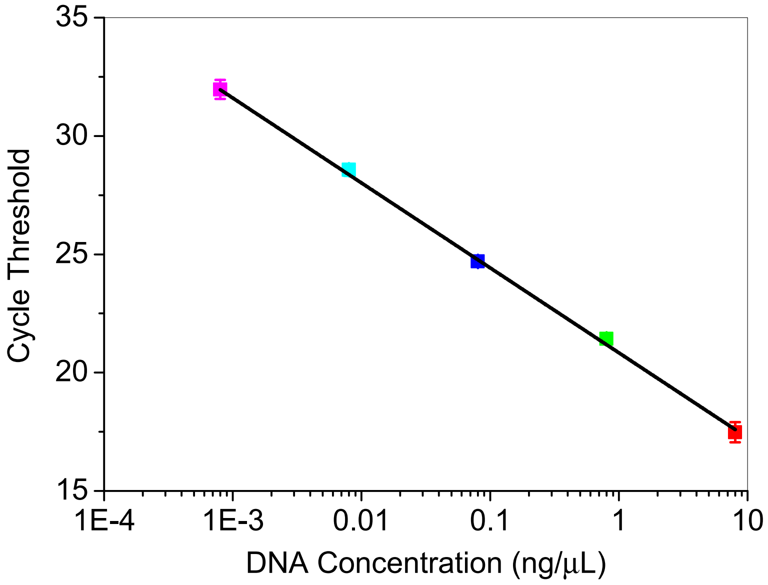

Table S2. Comparison between commercial and developed systems.

performance/ platform	Commercial PCR system (Applied Biosystems)	Commercial Sample preparation machine (Applied Biosystems)	Automated fast PCR system (in this paper)
Sample preparation time	-	~40 min / 1-13 samples	~5 min / 2 samples
Sample preparation method	-	membrane with centrifugation	magnet bead method
Cycles / completion time	40cycles / 38min	-	40cycles / 13.5min
Denature temperature	95±1℃	-	93±2℃
Annealing/elongation temperature	60±1℃	-	62±2℃
Extension time	10 sec	-	10 sec
Heating temperature	105℃	-	105℃
Cooling temperature	-	-	10℃
Heating temperature	2.5℃	-	10.2℃
Cooling temperature	2.5℃	-	16.5℃
System size	34cm(W) × 49 cm (H) × 41cm (D)	50cm(W) × 55 cm (H) × 57cm (D)	25cm(W) × 23 cm (H) × 18cm (D)
